# Deciphering the *Bacillus amyloliquefaciens* B9601-Y2 as a Potential Antagonist of Tobacco Leaf Mildew Pathogen During Flue-Curing

**DOI:** 10.3389/fmicb.2021.683365

**Published:** 2021-07-14

**Authors:** Zuxian Pan, Shahzad Munir, Yongmei Li, Pengbo He, Pengfei He, Yixin Wu, Yan Xie, Zongwei Fu, Yongzhan Cai, Yueqiu He

**Affiliations:** ^1^State Key Laboratory for Conservation and Utilization of Bio-resources in Yunnan, Yunnan Agricultural University, Kunming, China; ^2^Qujing Branch of Yunnan Provincial Tobacco Company, Qujing, China

**Keywords:** *Bacillus*, fungi, *Nicotiana tabacum*, *Rhizopus oryzae*, tobacco

## Abstract

Tobacco leaf mildew caused by *Rhizopus oryzae* (*Mucorales, Zygomycota*) is an important and devastating post-harvest disease during flue-cured tobacco period, and also is known to cause diseases of fruits and vegetables. In this study, assessment of several candidate biological control agents were first tested *in vitro* to determine their antifungal activities and potential strains were further applied to tobacco leaves to prevent pathogen colonization during the tobacco-curing process. *In vitro* screening of 36 bacteria and the isolates of one fungus were performed for their antifungal activities against *R. oryzae* using dual culture method. Potential five isolates viz. *Bacillus amyloliquefaciens* B9601-Y2 (Y2), *B. amyloliquefaciens* YN201728 (YN28), *Pseudomonas* sp. (Pb), and *B. amyloliquefaciens* YN201732 (YN32) and *T. harzianum* B (Th-B) from total screened isolates have shown remarkable results for controlling the mycelial growth of *R. oryzae*. Finally, out of these five isolates, *B. amyloliquefaciens* B9601-Y2 potentially reduced the mycelial growth of fungal pathogen with great inhibitory effect. In order to get a better understanding of the biocontrol effect of B9601-Y2 in a flue-curing barn, various suspension density tests with two application methods involving spraying and soaking were examined. Two application methods of *B. amyloliquefaciens* B9601-Y2 had 98.60 and 98.15% control effects, respectively. In curing barn, the incidence in the treatment group was significantly reduced and tobacco leaves did not get mildew. Altogether, the study demonstrated that candidate bacterial strain *B. amyloliquefaciens* B9601-Y2 is a potential antagonist for the management of tobacco leaf mildew during flue-curing.

## Introduction

Tobacco (*Nicotiana tabacum* L.) is one of the most important commercial crops widely cultivated in about 75 countries in many agricultural regions around the world. The tobacco industry achieved an annual output of 3 million tons in recent years (FAOSTAT, 2018). This crop is often used in cigarette blends or mixtures for pipe smoking and also a valuable medicinal plant as it has high alkaloid content. However, many post-harvest diseases such as frogeye leaf spot of tobacco (Dixon et al., 2018), blue mold (Blanco-Meneses et al., 2018), and tobacco soft rot (Wang R. et al., 2017) during production result in devastating losses. After harvesting from the field, leaves are stored for curing, redrying, aging, and fermentation, which negatively affect leaf carotenoids that slowly oxidize and importantly, cause degradation (Zhu et al., 2016; Popova et al., 2019).

During these processes, numerous diseases caused by phytopathogens such as *Aspergillus, Penicillium*, and *Rhizopus* reduce yields and monetary returns (Wang J. et al., 2017). *Rhizopus oryzae* causes leaf and petiole mildew and losses during flue-cured processing of tobacco leaves (Kortekamp et al., 2003; Wang et al., 2016; Pan et al., 2019). In Yunnan Province, China, the average annual loss due to diseases in curing barn is estimated to be 15–25% (Chen et al., 2020). Infection by *R. oryzae* usually begins at wound sites or dead cells and then gradually extends to leaves. Petiole surface covers with a dark brown mycelial mat of *R. oryzae*, resulting in decomposing and softening of internal tissues. The most characteristic symptoms of tobacco leaf mildew are a slimy, wet rot lesion that spreads from the rotting main petiole to the mid-veins and leaves, then appears as downy mold (Zheng et al., 2017). Disease outbreaks can occur rapidly and cause severe losses, especially under prolonged humid and warm conditions they may cause further losses through post-harvest infection in storage bins (Pan et al., 2019). Fungi that are air-borne and can produce a large number of spores, can survive for many years below 70°. The optimal growth temperature of *R. oryzae* ranges from 36.8 to 37.9° (Pan et al., 2019). This pathogen causes pre- and post-harvest crop losses worldwide, with a wide host range, such as banana (Kwon et al., 2012), peanut (Xu et al., 2015), sunflowers (Mathew et al., 2015; Zhou et al., 2018), sweet potato (Holmes and Stange, 2002; Wang J. et al., 2017), tomato (Khokhar et al., 2019) and yellow oleander (*Cascabela thevetia*) (Arif et al., 2017). The consequences of plant diseases range from major damage to minor nuisances. These problems have generated immense pressure and crisis on farmers to cut yield losses. Recently, several physical and chemical control measures have been used for the management of tobacco leaf mildew. Application of chemicals in the form of chlorothalonil, carbendazim, acid phosphorus, manganese, and zinc (Su et al., 2010); natural antimicrobials such as sodium d-isoascorbate, calcium salts, hydrogen peroxide, and azoxystrobin (Kortekamp, 2006) have been used to manage this disease. Different flue-cured tobacco varieties and the regulation of air flow in bulk curing barns (Popova et al., 2019) can also prevent and control mildew disease. However, synthetic chemicals can enhance crop productivity but also cause undesirable side effects, such as food contamination, environmental dispersal, and higher food production cost (Jiao et al., 2020). Owing to concerns about environmental contamination and harmful effects on non-target organisms, the demand for ecofriendly alternatives to chemical control has increased. Therefore, due to the widespread and refractory nature of the disease, no matter what flue-cured tobacco and storage technology is used, an alternative control of this disease is a serious challenge (Li et al., 2019; Zhang et al., 2020).

Biological control is an eco-friendly and cost-effective strategy, which can replace or integrate with chemical substances to achieve a high level of protection with sustainable production and also avoids the development of pathogen resistance (Cui et al., 2019; Jiao et al., 2020, 2021). This can be accomplished by protecting the host plant through the various biological activities of the bioagents by following appropriate application practices. Previous reports have shown that *Pseudomonas aeruginosa* as a biological control agent can effectively control tobacco bacterial wilt (TBW) and tobacco black shank (TBS) and even though it was found to be a good alternative to chemical fungicides (Ma et al., 2018) it has not been carried out in practice. The use of beneficial antagonists to control plant disease is a promising way to protect plants that can reduce the need for chemical substances.

In this study, we screened 37 isolates (36 bacteria and one fungus) for their antifungal activity against isolated *R. oryzae* by using dual culture and leaf disc assay. Antagonism—including biological control effect and microbial suspension is also studied here for disease management. We evaluated the ability of candidate biocontrol strains isolated from tobacco in the form of microbial suspension against leaf mildew disease caused by *R. oryzae*. In addition, we investigated candidate biocontrol strains against this pathogen under barn conditions on a large scale. Finally, the best strain *B. amyloliquefaciens* B9601-Y2 was used in different experiments to properly establish disease management strategies to maintain tobacco crop production with different application methods.

## Materials and Methods

### Tobacco Leaves and Microbial Strains

Rotten leaves of *Nicotiana tabacum* cv. Yunyan 105 were obtained from Geyi, Xuanwei, Yunnan Province, China in 2018 (Supplementary Figure 1). Tobacco leaves of susceptible cultivar Yunyan 105 with no signs of infection or apparent injury were also harvested from the same place (Xuanwei, Yunnan) during the commercial ripening period. Candidate microbes comprising 36 bacterial strains and 1 fungal strain (Supplementary Table 1), obtained in a previous study (Pan et al., 2019) and preserved in the Laboratory of Biocontrol and Plant Pathology (Yunnan Agricultural University, Kunming, China), were used in this study. Fungal pathogen *R. oryzae* was previously isolated from naturally infected tobacco leaves in Xuanwei (China). Luria Bertani broth (LB) and potato dextrose agar (PDA) culture media were used for the cultivation of bacteria and fungi, respectively.

### Synergism Assay for Candidate Microbial Strains

Synergism was assessed for all the combined strains tested on LB agar plates. Burkholder’s “spot-on lawn” method (Burkholder et al., 1966) was used to screen the synergistic effect of bacterial strains. Bacterial strains were obtained from the initial stock and pre-grown on LB plates at 37° for 48 h. Then a fresh colony of each strain was dissolved in sterile saline (0.85% NaCl). Strains were confronted with each other onto LB plates to obtain a full interaction of all strains with each other. The plates were incubated for 2-3 days at 37° to check the synergistic effect of strains. Bacterial strains grown together were used for further study.

### Inoculum Preparation of Candidate Microbes and Pathogen

Preserved candidate microbes were picked out by a sterile teasing needle and transferred to a fresh LB medium plate. The plate was incubated at 32°C for 72 h to allow bacterial growth. When a single bacterial colony appears, plates were kept at 4°C. A bacterial suspension was prepared by inoculating 100 mL of LB in Erlenmeyer flasks with a loopful of cells, and the flask was incubated on a rotary shaker at 180 rpm for 72 h at 37°C and diluted to different densities, and stored at 4°C.

For the pathogen, the method described by Sukhada et al. (2011) with minor modification was followed. Sterile filter paper discs with *R. oryzae* were cultured on a PDA medium plate containing 10 μg/mL chloramphenicol solution for 4 days. Plates were cultured at 32°C for 72 h for fungal sporulation. The 15 mL of sterile distilled water (SDW) was added to an inoculated plate to collect the spores/mycelia and the mixture was thoroughly ground. The spore suspension was carefully drawn, and adjusted to different concentrations through a hemocytometer (Sigma-Aldrich, China).

### Screening Candidate Microbes for Their Antifungal Activities

#### Dual-Culture Antagonism Assay

Biocontrol strains preserved in the laboratory were assessed for their ability to inhibit *R. oryzae* on PDA medium as described previously (Sukhada et al., 2011), with some modifications. *Rhizopus oryzae* spore suspension was adjusted to 10^4^ spores/mL with SDW and spores concentration was measured by a hemocytometer (Sigma-Aldrich, China). An aliquots of 100 μL spore suspension was spread on a fresh PDA plate and incubated at 32°C. After 5 h, a single candidate bacterial colony was stirred with a sterilized teasing needle and then immersed in four sites, approximately 2 cm away from the edge of the PDA plate. The plates were incubated in a growth chamber at 32 °C. Each bacterial isolate was tested three times. Antifungal potentials of *Trichoderma harzianum* and *T. viride* were evaluated as performed previously with slight modifications (Meena et al., 2017). Five days old culture of *T. harzianum* TH-B was used in PDA plates using the mycelial plugs (5 mm) in the center. In the same plates, tested pathogen *R. oryzae* was placed at the opposite side of the plates. The control plates consist of pathogen only without the application of biocontrol strains. After 2 days, the number of inhibition zones (IZ) between the fungus and the bacteria was recorded (An IZ > 0.2 cm indicated that the antagonist bacteria have an antifungal effect).

#### Tobacco Leaf Disc Interaction Test

In detached leaf disc assay, the healthy leaves of susceptible cultivar Yunyan 105 were chosen. The leaves were cut into small discs with a diameter of 1.5 cm and rinsed four times with SDW (1 min each disc), then left to dry at room temperature. Bacteria suspensions were diluted to 10^8^ CFU/mL by using the plate count method as performed previously (Munir et al., 2020). Fungal pathogen suspension was prepared as described above and the concentration was adjusted to 10^4^ spores/mL in SDW by using a hemocytometer with the addition of 0.1 g/L Tween 80. Both sides of leaf discs were sprayed by prepared spore suspension using a manual sprayer. The bacterial suspension was sprayed on both sides of the dried leaf disc and transferred to a petri dish, covered with a parafilm, and incubated in a growth chamber at 32°C for 5 h to make interaction. Bacteria were recovered from the leaf disc by washing the leaf disc with 1,000 μL of SDW. An aliquot of 200 μL of dilutions was spread on a PDA plate and incubated at 32°C for 3 days. The spore germination on the plate indicated the interaction between bacterial isolates and pathogens. The assay consisted of 10 leaf discs with three replicates.

### Screening of Selected Candidate Strains for Their Biocontrol Capacity Against Tobacco Leaf Mildew in Field Curing Barns

#### Effect of Different Inoculum Concentrations

Five (5) biocontrol isolates viz. *B. amyloliquefaciens* B9601-Y2 (Y2), *B. amyloliquefaciens* YN201728(YN28), *B. amyloliquefaciens* YN201732(YN32), *Pseudomonas* sp. (Pb), and *T. harzianum* B (Th-B) with potential antifungal effects in dual culture and disc interaction from the above experiments were further applied in the curing barn located at Shimo Village, Geyi Township, and Xuanwei City, Yunnan Province. Biocontrol bacteria and fungi were prepared according to the above methods. Among them, original concentrations of the bacterial strains viz. *B. amyloliquefaciens* B9601-Y2 (Y2), *B. amyloliquefaciens* YN201728(YN28), *B. amyloliquefaciens* YN201732(YN32), and *Pseudomonas* sp. (Pb) strains was 10^6^ CFU/mL, while *T. harzianum* B (Th-B) spore suspension concentration was 10^8^ spores/mL. All biocontrol agents were applied at the original concentration and concentration of 10 fold dilution.

After collecting tobacco leaves in the field, the petioles of 30∼40 cm were soaked in different concentrations and different types of biocontrol suspensions for 10 min. Fresh tobacco leaves were taken out and stretched to air-dry naturally, then flue-cured tobacco shall follow the normal flue-cured tobacco process. The control group was kept untreated. After 7 days, disease incidence was observed and recorded. According to the above method, the disease index and control effect was calculated. In this experiment, 10 bars of tobacco leaves (100 pieces/bar) were treated with each biocontrol suspension and the experiment was repeated 3 times.

#### Effect of Different Microbial Mixtures and Application Methods

Further, Y2, Pb, and Th-B were selected as mixed application strains. Y2, Pb bacterial suspension, and *Trichoderma* Th-B spore suspension were prepared according to the above method and two of them were mixed with 1:1 to prepare Y2+Pb, Y2+Th-B, Th-B+Pb suspension. The positive control is treated by soaking in single strain suspension, while negative control was treated with SDW without soaking. Healthy tobacco petioles were first washed with SDW, sterilized on the surface by soaking in 70% ethanol for 30 s, and rinsed with sterilized water 4 times (30 s each), then dried at room temperature. Pathogen spore suspension (10^4^ spores/mL) was sprayed onto the petiole surface. After air-drying, petioles were immersed for 10 min in single and mixed applications of different strains. The dried petioles were placed in sterilized petri dishes, sealed with plastic wrap to maintain high humidity, incubated at 32°C, and monitored routinely after every 24 h.

After the seventh day of observation, the disease grade scale (DG) was categorized from 0 to 9 based on lesion area: (0) no symptoms; (1) lesion area smaller than 5% of the total petioles area; (3) 5–25% of petioles area infected; (5) 25–50% of petioles area infected; (7) 50–75% of petioles area infected; (9) larger than 75% infected area.

The disease severity was assessed by disease rate (DR) and disease index (DI) (Alahmad et al., 2018), and relative control effect (RCE) is used to assess resistance:

$$\text{DI}=\frac{\sum \left(D\,G_{i}\times n_{i}\right)\times 100}{n=D\,G_{m\,a\,x}};\text{DR}=\frac{m}{n}\times 100\%;\text{RCE}=\frac{D\,I_{c}-D\,I_{i}}{D\,I_{c}}\times 100\%$$

Where DG_*max*_ is the maximum value of disease grades, n_*i*_ is the number of petioles with each disease grade, m is the total number of rot petioles, n is the total number of petioles observed, DI_*c*_ is the value of the control disease index. The 10 bars of tobacco leaves (100 pieces/bar) were treated with each biocontrol suspension and the experiment was repeated 3 times.

#### Evaluation of the Biocontrol Effect of Different Inoculum Concentrations of *Bacillus amyloliquefaciens* Strain B9601-Y2 Under Laboratory Conditions

The antifungal activity of highly effective strain *B. amyloliquefaciens* B9601-Y2 (Y2) at a different concentration was tested against *R. oryzae* on tobacco petiole. The bacterial suspension was prepared as described above. The density of biocontrol bacteria culture was adjusted to 10^5^, 10^6^, and 10^7^ CFU/mL with SDW. Fungal spore inoculum was prepared by mixing 10^4^ spores/mL spore suspension. Healthy tobacco petioles were first washed as described above. Spore suspension (10^4^ spores/mL) was sprayed onto the petiole surface for 10 min, as mentioned above. After 3 days of observation, DI, DR, and REC were calculated. The concentration of suspension of each biological agent consisted of 10 petioles with 3 replicates for the confirmation of results.

### Evaluation of the Biocontrol Effect of *Bacillus amyloliquefaciens* Strain B9601-Y2 in Field Curing Barns

#### Effect of Different Inoculum Concentrations

In 2018, pre-selected *B. amyloliquefaciens* Y2 was screened for its ability to reduce tobacco leaf mildew at Shimo Village, Geyi Township, and Xuanwei City, Yunnan Province. The cultivation of this crop followed the standard agronomic practices. Fresh tobacco leaves were soaked 40-50 cm, the petiole was covered with the fermentation broth of Y2 at a concentration of 10^5^, 10^6^, and 10^7^ CFU/mL for 10 min before roasting. The fourth without treatment was kept as a control to assess the disease. Treatments in each curing barn consisted of 3 replicates in a completely randomized block design. Each experiment was carried out three times. Disease evaluation followed the same procedure as above.

#### Effect of Different Application Methods

Effective strains were selected after *in vitro* and *in vivo* bioassay, to evaluate their effectiveness under field conditions against tobacco leaf mildew during curing at Seka Village, Reshui Township, Yunnan Province. Standard agronomic practices were followed in crop cultivation. Fresh tobacco leaves were soaked (300 leaves for each concentration) in a suspension of Y2 at 10^5^ CFU/mL or sprayed (1,000 leaves for each treatment) on the tobacco petiole top until the drop dripped. An untreated control was used as a negative test. In a completely randomized block design, treatments in each curing barn involved three replicates. Disease assessment was performed as described above after 7 days. Each experiment was carried out three times. Disease evaluation followed the same procedure as above.

#### Statistical Analysis

The experiment was carried out in a completely randomized design. All data were subjected to analysis of variance (ANOVA) at a 0.05 significance level, using the SPSS version 22.0 (SPSS Inc., Chicago, IL). Duncan’s multiple range test was applied to separate means. All data are expressed as the mean ± SDs from independent experiments.

## Results

### Lab Screening of Candidate Bacteria Against Fungal Pathogen

#### Antifungal Activity *in vitro*

**FIGURE 1:**
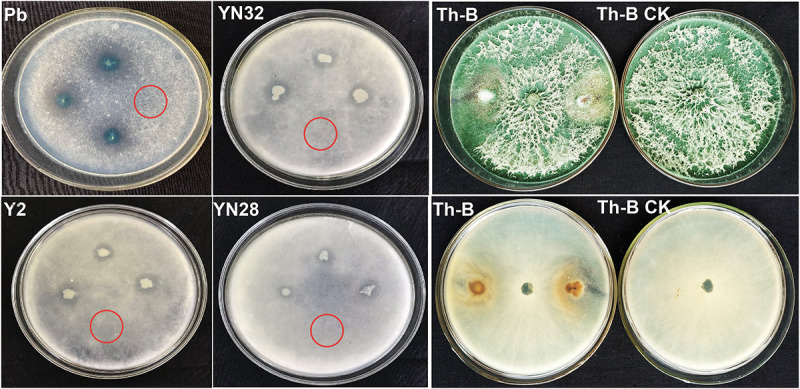
Lab screening of antagonist against fungal pathogen. Biocontrol agents with different inhibitory effects against *Rhizopus oryzae*. *Pseudomonas* sp. (Pb), *B. amyloliquefaciens* YN201732 (YN32), *B. amyloliquefaciens* B9601-Y2 (Y2), and *B. amyloliquefaciens* YN201728 (YN28) were tested *in vitro*. *Trichoderma* (Th-B) displaying antifungal activity against fungal pathogen, which occupied all the space in the plate. Inverted section of Th-B showing that the candidate strain antagonizes the pathogen. The red circle indicates that the control treatments showed no inhibition.

Thirty-seven (37) microbial strains from our previous study (Pan et al., 2019) were inoculated on ϕ9cm PDA plates spread with 100 μL spore suspension of *R. oryzae*. All selected bacterial strains were also tested against each other to check synergism (Supplementary Figure 2). The results showed that most of the strains can grow easily with each other. Four (4) bacterial strains Y2, Pb, YN28, YN32, and a fungal strain *T. harzianum* Th-B showed antagonistic potential against *R. oryzae*. Initially, these five candidate isolates against *R. oryzae* were used to test *in vitro* on tobacco leaf discs. However, the antifungal effect of biocontrol bacteria was not obvious in the plate confrontation experiment (Figure 1). *Bacillus amyloliquefaciens* Y2 showed a very good antifungal effect against *R. oryzae* through the growth of mycelia on the PDA plates (Supplementary Table 1) and revealed a better antagonistic effect compared to other strains by involving a maximum reduction in leaf disc assay.

#### Tobacco Leaf Disc Interaction Test

**FIGURE 2:**
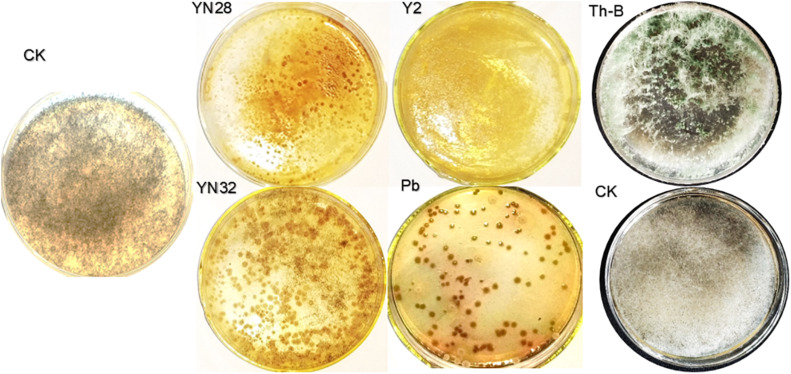
Tobacco leaf disc interaction test. Both sides of leaf discs were sprayed by prepared spore suspension using a manual sprayer. Spore suspension of the pathogen and candidate biocontrol strains brought in interaction in the growth chamber for 5 h. An aliquot of 200 μL of dilutions was spread on a PDA plate and incubated at 32°C for 3 days.

According to the screening experiment, four strains of bacteria Y2, Pb, YN32, YN28 and one strain of *Trichoderma* Th-B have a certain inhibitory effect on the growth of fungal pathogen (Figure 2). Therefore, these five candidate strains were selected for the leaf disc experiment. After a period of moisturizing culture, the candidate strains on the leaf disc surface were eluted, and the plate was cultivated. Compared with the control group, *R. oryzae* in the eluate of leaves treated with Y2, Pb, and YN28 no longer grow. It shows that Y2, Pb, and YN28 have obvious inhibitory effects on the growth of the *R. oryzae* pathogen, and Y2 has the best inhibitory effect. *Bacillus amyloliquefaciens* YN32 and *T. harzianum* Th-B can also inhibit the growth of the fungal pathogen in the eluate. Therefore, these five candidate strains were selected for further field control experiments.

### *In vivo* Evaluation of the Antifungal Activity Against *Rhizopus oryzae*

#### Confirmation of the Preventive Effect Under Curing Barn Conditions

Based on the above experimental results, the five strains were selected to verify the control effect in the curing barn. Mature tobacco leaves with petiole were collected from the field and soaked in different concentrations of various suspensions of microbial strains. Tested strains significantly affected disease incidence and *R. oryzae* in the same way as reported in the lab. *Bacillus amyloliquefaciens* Y2 and *Trichoderma* strain Th-B had a good control effect on tobacco leaf mildew. The strains YN28 and YN32 displayed a good control effect but these strains reduced the quality of cured tobacco leaves. After curing, the leaves soaked in YN28 and YN32 were generally green, which greatly reduced the quality of tobacco leaves. Therefore, YN28 and YN32 bacterial strains were no longer used in the following experiments (Table 1).

**TABLE 1:** Effect of various concentrations of five treatments on tobacco mildew disease.

Isolate	Concentration (CFU/mL)	Disease index	Disease rate (%)	Control efficacy (%)
Control	NA	19.64 ± 0.09a	43.12 ± 0.77a	NA
Y2	10^6^	0.04 ± 0.04b	0.43 ± 0.43b	99.80 ± 0.20d
	10^5^	0.25 ± 0.07bc	2.75 ± 0.77c	98.74 ± 0.35bcd
Pb	10^6^	0.10 ± 0.06b	1.16 ± 0.72bc	99.47 ± 0.33cd
	10^5^	0.26 ± 0.12bc	2.90 ± 1.31c	98.67 ± 0.60bcd
YN28	10^6^	0.11 ± 0.04b	1.21 ± 0.40bc	99.45 ± 0.18cd
	10^5^	0.24 ± 0.06bc	2.67 ± 0.67b	98.78 ± 0.31bcd
YN32	10^6^	0.14 ± 0.06b	1.61 ± 0.64bc	99.27 ± 0.29bcd
	10^5^	0.29 ± 0.09bc	3.18 ± 0.96c	98.55 ± 0.44bc
Th-B	10^8^	0.36 ± 0.06bc	4.06 ± 0.70c	98.15 ± 0.32b
	10^7^	0.67 ± 0.09c	7.45 ± 0.97cd	96.59 ± 0.44a

**Data are mean ± SD. Different letters after significant difference at p < 0.05 level by Duncan’s test.**

#### Biocontrol Effect With Different Application Methods

The above three screened strains viz. *B. amyloliquefaciens* B9601-Y2 (Y2), *Pseudomonas* sp. (Pb), and *T. harzianum* B (Th-B) were tested for mixed control. The results showed that both mixed and single application had higher than 95% control effects and the differences in control effects between different treatments were not significant (Table 2). Initially, the experiment was carried out using individual strains through a spray method and also by a combination of strains in soak methods. We found that spraying with individual strains can effectively control the disease, so further experiments were not carried out with combination during the spraying method. A low dose of candidate strain was used in curing the barn.

**TABLE 2:** Effect of different application methods on tobacco mildew.

Application method	Isolate	Disease index	Disease rate (%)	Control efficacy (%)
Spray	Control	21.24 ± 0.23a	61.33 ± 0.91a	NA
	Y2	0.48 ± 0.16b	4.28 ± 1.45b	97.76 ± 0.76e
	Pb	1.08 ± 0.04b	9.69 ± 0.35bc	94.93 ± 0.18cd
	Th-B	1.78 ± 0.26bc	16.01 ± 2.31c	91.62 ± 1.21a
Soak	Control	21.18 ± 0.11a	59.22 ± 0.52a	NA
	Y2	0.54 ± 0.11b	4.88 ± 1.02b	97.45 ± 0.53e
	Pb	0.89 ± 0.05b	8.02 ± 0.44b	95.81 ± 0.23de
	Th-B	1.79 ± 0.13bc	16.14 ± 1.19c	91.56 ± 0.62a
	Y2/Pb	1.67 ± 0.22bc	15.07 ± 1.94c	92.12 ± 1.02ab
	Th-B/Pb	1.46 ± 0.12b	13.16 ± 1.06bc	93.12 ± 0.56abc
	Y2/Th-B	1.22 ± 0.05b	11.02 ± 0.46bc	94.24 ± 0.24bcd

**Data are mean ± SD. Different letters after significant difference at p < 0.05 level by Duncan’s test.**

#### Effect of Various Concentrations of Bacterial Suspensions

**FIGURE 3:**
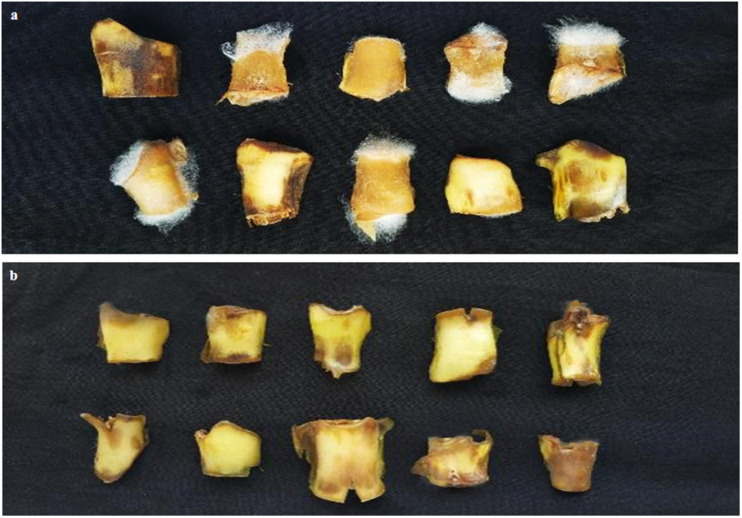
Effect of *B. amyloliquefaciens* Y2 was used as a biocontrol agent against *R. oryzae in vivo*. **(a)** The control showed white hair like mycelium of the fungal pathogen; **(b)** treatment with *B. amyloliquefaciens*Y2 indicated the inhibition of pathogen). The experiment was repeated three times.

We found that candidate strain *B. amyloliquefaciens* B9601-Y2 displayed the best antifungal activities against the fungal pathogen in different assays, therefore, it was chosen for further experiments. Briefly, spore suspension of *R. oryzae* and candidate strain *B. amyloliquefaciens* B9601-Y2 suspension were sprayed onto sterilized fresh tobacco discs, respectively. At the same time, the pathogen was sprayed on tobacco discs, which served as a control group. Leaves became softened and gradually spread in later stages and white hair-like mycelium appeared out of the wound. Tobacco discs were rotten and the mycelium stuck to the surface of the disc (petiole) due to high humidity. Diseased tissue emitted mildew with an odor smell and DI is 53.33%. The lesions formed in a treatment group approximately 1 day later than the control group. The infection rate was lower and the disease index was significantly reduced with higher than 95% control efficacy (Figure 3), indicating that *B. amyloliquefaciens* B9601-Y2 has potential in the prevention and control of tobacco leaf mildew. The disease indexes at the concentrations of 10^5^, 10^6^, and 10^7^ CFU/mL of Y2 strain were 2.59, 1.48, and 0.00, respectively. The control effects are 95.14, 97.22, and 100.00%, respectively, indicating that the effect of increasing concentration on the control effect is not obvious (Table 3).

**TABLE 3:** Effect of *Bacillus amyloliquefaciens* Y2 on tobacco mildew in laboratory.

Site	Application	Concentration (CFU/mL)	Disease index	Disease rate (%)	Control efficacy (%)
Laboratory	Soak	Control	53.23 ± 0.23a	85.00 ± 1.64a	NA
		3.2 × 10^7^	0.00 ± 0.00b	0.00 ± 0.00b	100 ± 0.00a
		3.2 × 10^6^	1.48 ± 0.64bc	13.33 ± 5.77c	97.22 ± 1.15ab
		3.2 × 10^5^	2.59 ± 0.64c	23.22 ± 5.77d	95.14 ± 1.15b

**Data are mean ± SD. Different letters after significant difference at p<0.05 level by Duncan’s test.**

### Large-Scale Evaluation of Y2 in Curing Barn

#### Effect of Different Concentrations on the Control Effect in Curing Barn

**FIGURE 4:**
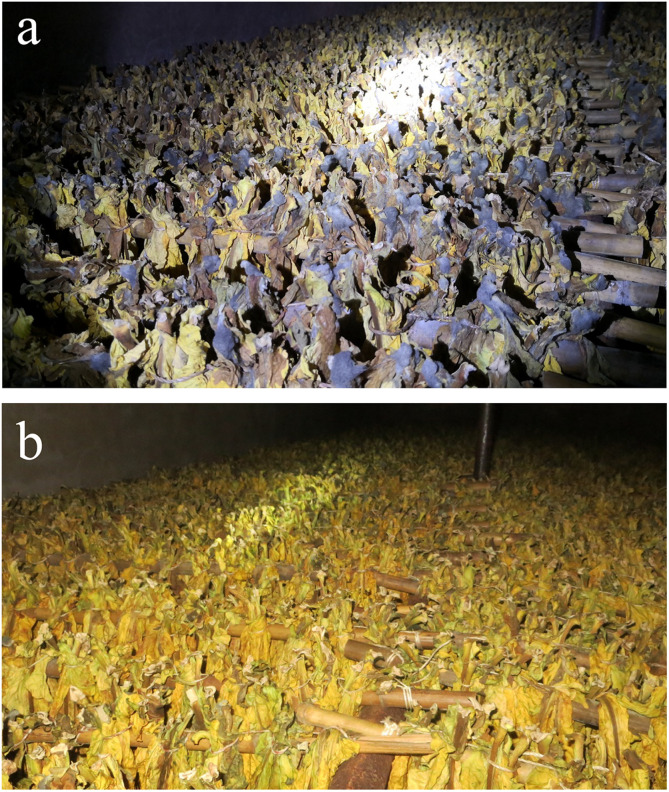
Inhibition of pathogen in curing barn. The experiment was conducted in field conditions naturally infested with fungal pathogen *R. oryzae*. **(a)** Infested tobacco leaves with *R. oryzae.* **(b)** Effect of *B. amyloliquefaciens*Y2 was used as a biocontrol agent against *R. oryzae* under field condition.

To clarify the effect of concentration, the experiment was conducted in a curing barn naturally infested with *R. oryzae* in 2018 at Geyi Township, Xuanwei City, Yunnan. In the control group, initially, water-stained spots at the petiole of tobacco appeared, then become brown and rotten. Diseased parts were shrunk and softened and then extended from the base along the leaf vein to the tip of the leaf with a large number of white hyphae on the surface. The mycelium later became gray/black and eventually moldy and decayed tobacco leaves started expanding. However, the incidence in the treatment group was significantly reduced and the tobacco leaves did not get rot (Figure 4). Fewer leaves were affected by mold and the disease level was low. The control effect of all treatments reached as high as 98%, but there was no significant difference between groups (Table 4).

**TABLE 4:** Effect of *Bacillus amyloliquefaciens* Y2 on tobacco rot in curing barns (Shimo, Yunnan and Seka, Yunnan) experiments.

Site	Application	Concentration (CFU/mL)	Disease index	Disease rate (%)	Control efficacy (%)
Shimo	Soak	Control	20.95 ± 0.04a	42.12 ± 0.71a	NA
		1.5 × 10^7^	0.04 ± 0.06b	0.33 ± 0.58b	99.82 ± 0.58a
		7.5 × 10^6^	0.22 ± 0.11bc	2.00 ± 1.00c	98.95 ± 0.58ab
		1.5 × 10^5^	0.30 ± 0.06bc	2.67 ± 0.58c	98.60 ± 0.58ab
Seka	Spray	Control	31.35 ± 0.43a	51.33 ± 0.34a	NA
		1.1 × 10^5^	0.58 ± 0.37b	5.00 ± 2.94b	98.15 ± 1.23

**Data are mean ± SD. Different letters after significant difference at p < 0.05 level by Duncan’s test.**

#### Effects of Different Application Methods on the Control Effect in the Field Curing Barn

*Bacillus amyloliquefaciens* Y2 had a satisfactory effect for controlling tobacco leaf mildew. In order to understand the effect of application methods on results, the experiment was conducted in a curing barn naturally infested with *R. oryzae* in 2018 at Reshui Township, Xuanwei City, Yunnan. Two application methods, spraying, and soaking, had 98.60 and 98.15% control effect, respectively, showing that there was no significant difference between the two methods (Table 4).

## Discussion

Tobacco leaf mildew is commonly found throughout China where the pathogen *Rhizopus oryzae* can infect petioles and leaves during the flue-curing process with typical symptoms emerging at a later stage. Several physical and chemical control measures have been used to inhibit the growth of *R. oryzae* on tobacco leaves (Amadioha, 2001; Amadioha and Markson, 2007; Chaurasia et al., 2017; Uyar and Uyar, 2019). However, fumigation with chemicals can cause undesirable side effects, such as product contamination, environmental diffusion, and increase the production cost of goods. Despite the urgent need for effective and environmentally friendly alternatives, research is limited due to the lack of plant screening systems. To date, no significant measures have been taken to control this disease. A total of 37 candidate microbes were identified and all of these were tested to check for synergism against each other. The antagonists with inhibitory effects on each other were considered as outliers. We established that *B. amyloliquefaciens* B9601-Y2 is an effective biological control agent when used to soak or spray tobacco petioles in the curing barn, thereby significantly reducing the disease by 90% or above. Our previous study proved that this strain can tolerate high temperatures, i.e, temperature-tolerant during curing barn makes it one of the suitable candidates to control tobacco leaf mildew caused by *R. oryzae* (Pan et al., 2019).

*Rhizopus* has been a serious threat to a wide variety of foods stuff, fruits, and crops in agricultural and urban environments in many tropical and subtropical regions. The use of beneficial antagonistic microorganisms is a promising method of protecting plants from air-borne pathogens, which can reduce the need for chemical fungicides (Cartwright and SpurrJr., 1998; Sukhada et al., 2011; Widmer, 2014). Several microorganisms have been studied to inhibit the growth of *Rhizopus*. *Pichia membranefaciens* had a significant biocontrol effect against *Rhizopus stolonifer* on nectarine fruits, with an incidence rate of 60% reduction (Qing and Shiping, 2000). Zahavi et al. (2000) found two isolates, *Candida guilliermondii*, and *Acremonium cephalosporium* can control decay in grapes caused by *R. stolonifera* (Zahavi et al., 2000). Six antagonistic agents viz. *Trichoderma asperellum, T. atroviride, T. fasciculatum, T. harzianum*, *T. viride*, and *T. virens* have been found to manage the *R. oryzae* pathogen of tomato rot (Alka and Prajapati, 2017). Moreover, disease severity in TBW and black shank significantly reduced by *Pseudomonas aeruginosa* (Ma et al., 2018). In addition, three *P. fluorescens* isolates were used to control the postharvest disease of apple (Wallace et al., 2017) and another strain *P. chlororaphis* to control *Ceratocystis fimbriata* in postharvest sweet potatoes (Zhang et al., 2019).

Similarly, in this study, *T. harizanium* Th-B and *Pseudomonas* (Pb) also showed growth inhibition of *Rhizopus* on culture media and tobacco leaf discs, but their antifungal interactions or potential control effect were not examined in large-scale on plants. Therefore, further large-scale research on the biocontrol effect of Th-B and *Pseudomonas* Pb will be needed.

This study started with *in vitro* analyses of the antifungal ability of 37 isolates against *R. oryzae*. Five remarkable isolates showed strong antagonistic activity and were further found to be effective in inhibiting the growth of *R. oryzae* in curing barn. In plant assays, we showed that before the establishment of *R. oryzae* infection, better biological control efficiency can be obtained by spraying B9601-Y2, which provides prospects for practical applications during flue-curing. In addition, *T. harzianum* Th-B and *Pseudomonas* Pb also showed significant inhibitory effects against *R. oryzae* in dual-culture and tobacco leaf disc assays, indicating their potential as biocontrol agents. Moreover, isolates YN32 and YN28, isolated from tobacco seeds, can also reduce disease incidence, but also delay the yellowing process which is considered to be a decline in quality. The study also tested whether a single strain or multiple strains of biocontrol agents can better control tobacco leaf mildew in the curing barn. The results showed that there was no significant difference between the two methods. Using mixtures of strains compared to using single strains may increase the chances of host colonization. The polymicrobial approach plays a significant role in excluding devastating pathogens (De Vrieze et al., 2018). The role of using each strain in combination is important to explore the properties of individual strain (Lindemann et al., 2016). Using a single strain or combination of strains depends mainly on the performance and target pathogen. A consortium of *Trichoderma* and *Pseudomonas* displayed significant effects against blast disease, but the effectiveness was reduced against blight disease (Jambhulkar et al., 2018). *Pseudomonas simiae* and *P. aeruginosa* strains have emerged as potential candidates in agriculture for disease control (Ma et al., 2018; Pieterse et al., 2020). Plant diseases can be managed efficiently through *P. aeruginosa*, which displayed excellent root colonizing ability and produced a wide range of metabolites during abiotic and biotic stress (Illakkiam et al., 2013). The *P. aeruginosa* NXHG29 was also reported for its excellent biocontrol ability against wilt disease caused by *Fusarium oxysporum* f.sp. *cubense* on banana (Li et al., 2012). In addition, a range of soil-borne pathogens has been successfully controlled through *P. aeruginosa* (Singh et al., 2010; Illakkiam et al., 2013). Apart from beneficial properties, this strain must be handled with much care as this strain is also an opportunistic pathogen and may lead to bloodstream, skin and soft tissue infections, and pneumonia (Driscoll et al., 2007). Cyanide production by *Pseudomonas* sp. causes an inhibitory effect on plant growth (Saharan and Nehra, 2011; Parikh and Jha, 2012), for example in lettuce and barnyard grass (Kremer and Souissi, 2001), *P. fluorescens* suppressed the emergence of green foxtail (*Setaria viridis* L.) (Daigle et al., 2002).

A 4 year field study on *Botrytis cinerea* in grapevines using a consortium of strains showed that a combination does not work to control disease, but *B. subtilis* or *Aureobasidium pullulans* alone is effective (Pertot et al., 2017). Another important factor to be considered is the strain used to control a particular disease, i.e., isolation source, available in a culture collection or a commercial strain. Different strains always have a differential mode of action against pathogens (Hunziker et al., 2015).

In this study, the above three isolates were evaluated as potential biocontrol agents against *R. oryzae*. However, antagonists for commercial use must fulfill different requirements. In addition to dealing with specific target plant pathogens, they must have appropriate market size, ecological characteristics, production costs, safety, environmental risks, and the possibility of intellectual property protection (Köhl et al., 2011). On the one hand, only *B. amyloliquefaciens* B9601-Y2 has been studied for safety and ecology, while strain B9601-Y2 has been patented and commercially used. Hence, *B. amyloliquefaciens* B9601-Y2 was finally selected for large-scale evaluation in curing barn trials. The genus *Bacillus* is known to inhibit a variety of bacterial, fungal, and fungal-like pathogens (Belbahri et al., 2017; Liu et al., 2018). Many studies have reported that members of the *Bacillus* act as elicitors for the induction of systemic resistance as well as plant growth promoters (Ramírez and Kloepper, 2010; Dinesh et al., 2015; Shao et al., 2015; Islam et al., 2016; Borriss, 2020). The ability to compete with the pathogen is related to the production of secondary metabolites (Boottanun et al., 2017; Chen et al., 2019) that possess antimicrobial activities (Alenezi et al., 2015, 2016, 2017; Mefteh et al., 2017), competition for essential nutrients (Wu et al., 2012), or stimulation of the host plant immune system (Chowdhury et al., 2015).

*Bacillus amyloliquefaciens* B9601-Y2, isolated from the rhizosphere of wheat in North China, has been found to inhibit a broad spectrum of pathogenic fungi; promote growth and rooting of crops and vegetables; improve the drought tolerance of wheat, corn, and broad bean; reduce the number of nematodes in tomato and tobacco roots and colonize many crops (Hao et al., 2012; He et al., 2013; Scholz et al., 2014; Cui et al., 2019). The antifungal lipopeptides bacillomycin D, fengycin, siderophore bacillibactin, and dipeptide bacilysin are produced by Y2 (He et al., 2013) and could be possible disease suppressive compounds against *R. oryzae*. The most active antifungal lipopeptides reported in this strain were bacillomycin D and fengycin. These compounds are responsible for suppressing spore germination and mycelium of pathogenic fungi such as *Fusarium oxysporum*, *Monilinia fructicola*, and *Rhizoctonia solani* (Koumoutsi et al., 2004; Liu et al., 2011). It has also been used as biocontrol of numerous plant diseases caused by *Bipolaris maydis*, *Fusarium verticillioides* (Cui et al., 2015), *Cochlioboluscarbonum*, *Curvularia lunata*, and *Exserohilum turcicum* (Xie et al., 2017), *Botrytis cinerea, Colletotrichum* spp., *Rhizoctonia solani*, and *Sclerotinia sclerotiorum* (Wu et al., 2012). In the present study, *B. amyloliquefaciens* B9601-Y2 was demonstrated as a successful biological control agent against tobacco leaf mildew disease caused by *R. oryzae* by soaking and spraying during the tobacco flue-curing process. Both of methods, spraying and soaking, had 98.60 and 98.15% control effect, respectively, showing that there was no significant difference between the two methods. Soaking is the conventional delivery system, using biocontrol agents to manage plant diseases. Both of these methods might play a role in antibiosis, competition, induction of resistance, and parasitism in targeting surface pathogens only. The timing of bacterial spray is an important factor that can facilitate the development of successful applications against tobacco pathogens.

## Conclusion

In summary, *R. oryzae* is an important fungal pathogen causing post-harvest tobacco disease during the flue-cured tobacco period and causes severe economic losses to the most economically valuable non-food crops in the world. *Bacillus amyloliquefaciens* B9601-Y2 effectively reduced the pathogen *in vitro* and under curing barn conditions on a large scale, implying it could successfully reduce the use of pesticides in tobacco crops. We conclude that both methods of spraying and soaking tobacco leaves in a biocontrol agent do not show much difference and a significant control effect was found when it was tested against pathogen *R. oryzae*. In addition, more research is needed to understand the most important mechanisms of *B. amyloliquefaciens* B9601-Y2 that can help to control this pathogen.

## Data Availability Statement

The original contributions presented in the study are included in the article/Supplementary Material, further inquiries can be directed to the corresponding author/s.

## Author Contributions

YH, YW, and YC conceived and designed the study and experiments. ZF, SM, YL, PBH, YX, and PH performed the experiments. ZF, YL, and PH analyzed the data. ZF, SM, and YH wrote the manuscript. All authors contributed to the final draft of the manuscript.

## Conflict of Interest

The authors declare that the research was conducted in the absence of any commercial or financial relationships that could be construed as a potential conflict of interest.

## Supplementary Material

Supplementary Figure 1 Tobacco petioles showing symptoms in field conditions. **(A,B)** Petioles with *Rhizopus oryzae* infection are indicated by an arrow pointing to the middle.

Supplementary Figure 2 Synergism assays for different bacterial strains. Red circle indicating two bacterial strains used together.

Supplementary Table 1 Candidate microbial strains used in the study.
