# Supplementary figures and images for: Deciphering the Bacillus amyloliquefaciens B9601-Y2 as a Potential Antagonist of Tobacco Leaf Mildew Pathogen During Flue-Curing
Source: Front Microbiol. 2021 Jul 14;12:683365. doi: 10.3389/fmicb.2021.683365 (PMC8317063; doi:10.3389/fmicb.2021.683365)

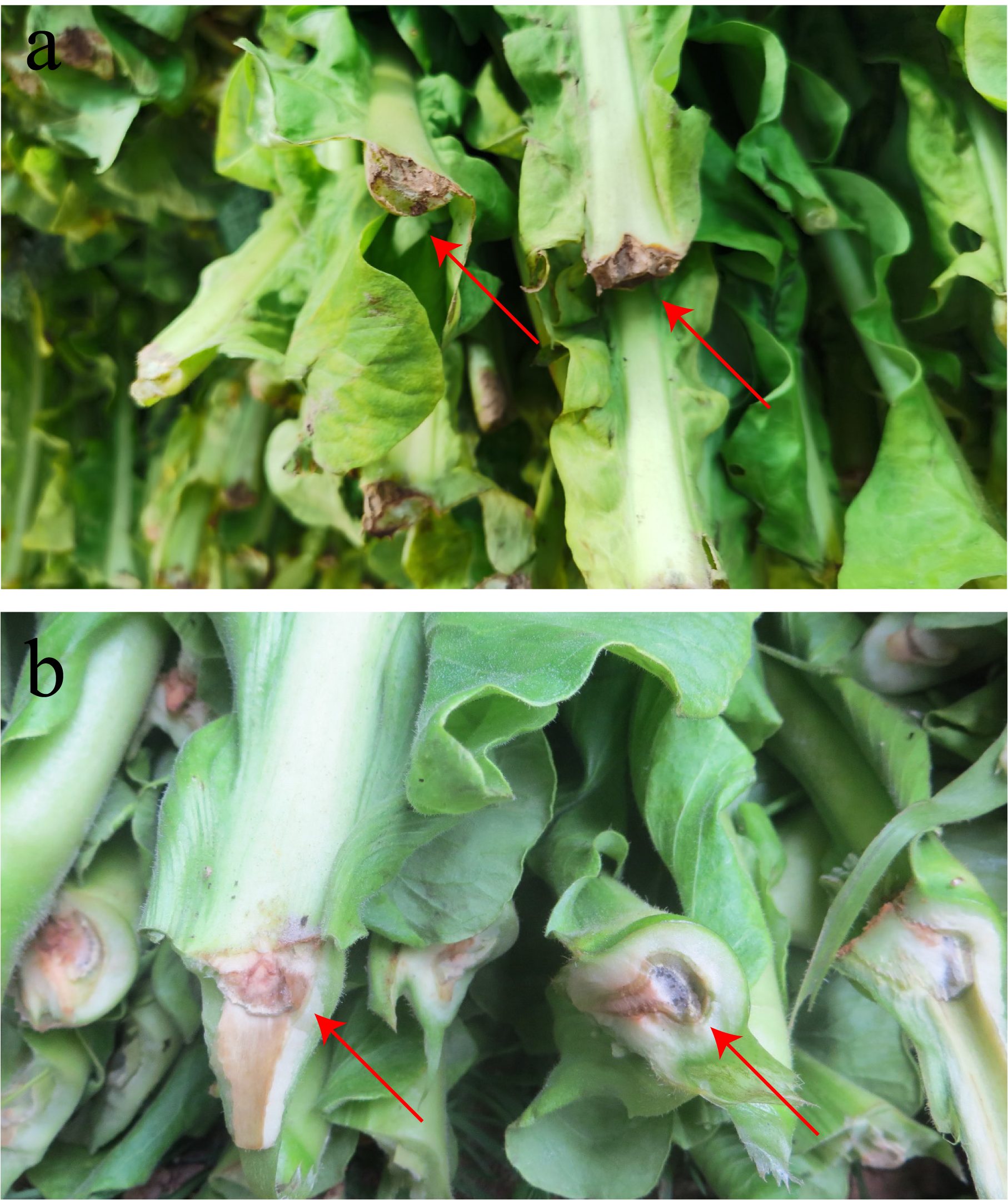

Supplement: Supplementary Figure 1 — Tobacco petioles showing symptoms in field conditions. (A,B) Petioles with Rhizopus oryzae infection are indicated by an arrow pointing to the middle. [file Image_1.TIF]

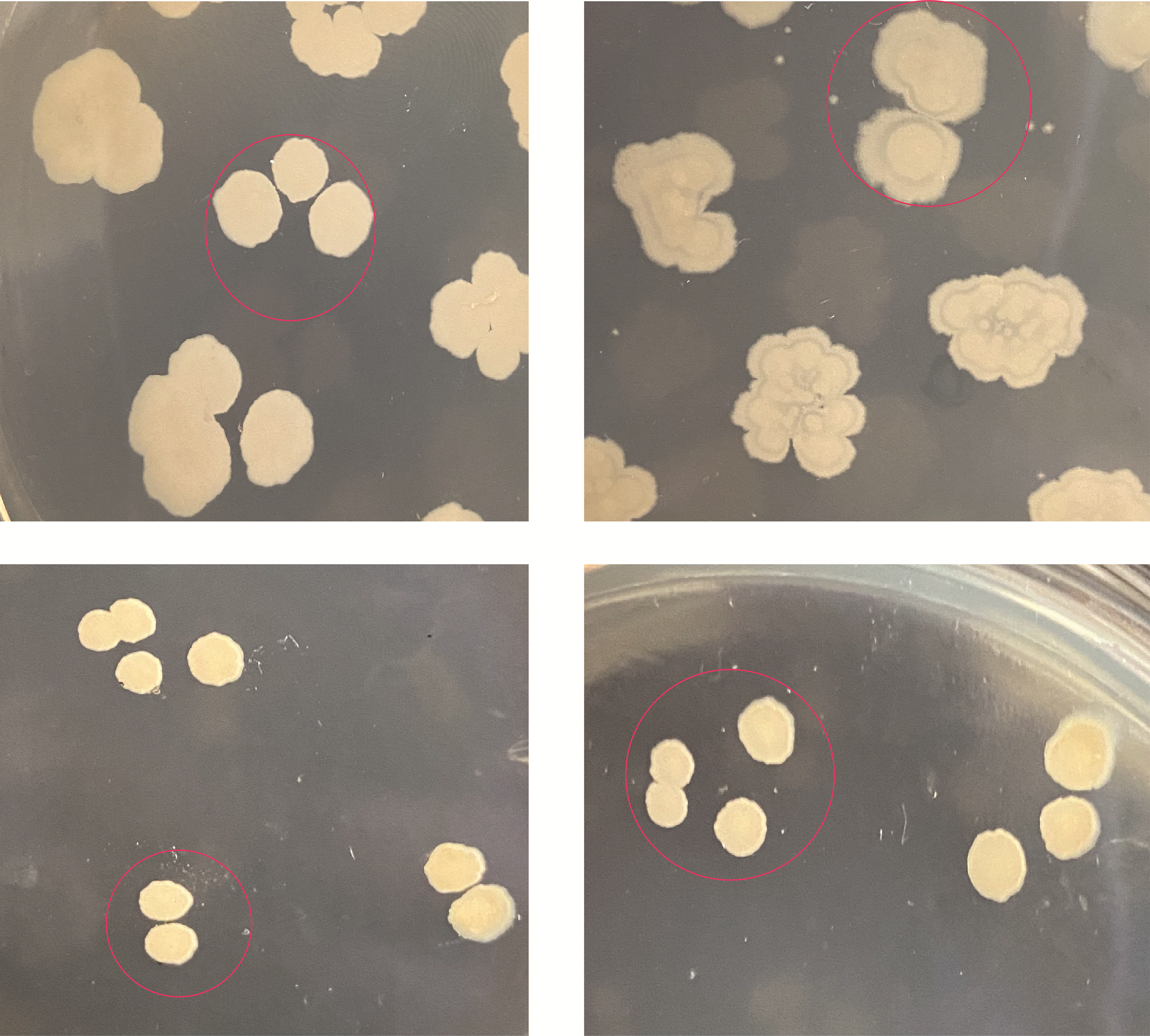

Supplement: Supplementary Figure 2 — Synergism assays for different bacterial strains. Red circle indicating two bacterial strains used together. [file Image_2.TIF]
