# Supplementary material for: Deciphering the Bacillus amyloliquefaciens B9601-Y2 as a Potential Antagonist of Tobacco Leaf Mildew Pathogen During Flue-Curing
Source: Front Microbiol. 2021 Jul 14;12:683365. doi: 10.3389/fmicb.2021.683365 (PMC8317063; doi:10.3389/fmicb.2021.683365)
Supplement: Supplementary Table 1 — Candidate microbial strains used in the study. [file Table_1.DOCX]

**Table S1.** Candidate microbial strains used in the study

| **S. No** | **Strain** | **Identification** | **Origin** | **Inhibition rate*** |
| --- | --- | --- | --- | --- |
| 1 | A27 | *Bacillus amyloliquefaciens* | Tobacco seeds | 0.00% |
| 2 | A28 | *Bacillus amyloliquefaciens* | Tobacco seeds | 83.33% |
| 3 | A32 | *Bacillus amyloliquefaciens* | Tobacco seeds | 100.00% |
| 4 | A9 | *Bacillus amyloliquefaciens* | Tobacco seeds | 0.00% |
| 5 | A16 | *Brevibacillus brevis* | Tobacco seeds | 0.00% |
| 6 | A37 | *Bacillus amyloliquefaciens* | Tobacco seeds | 0.00% |
| 7 | A2 | *Bacillus velezensis* | Tobacco seeds | 0.00% |
| 8 | O1 | *Bacillus amyloliquefaciens* | Sugarcane root | 0.00% |
| 9 | O2 | *Bacillus amyloliquefaciens* | Sugarcane root | 10.00% |
| 10 | P1 | *Bacillus amyloliquefaciens* | Sugarcane root | 0.00% |
| 11 | P2 | *Bacillus amyloliquefaciens* | Sugarcane root | 0.00% |
| 12 | P3 | *Bacillus amyloliquefaciens* | Sugarcane root | 0.00% |
| 13 | P4 | *Bacillus amyloliquefaciens* | Sugarcane root | 0.00% |
| 14 | Q7 | *Paenibacillus polymyxa* | Sugarcane root | 0.00% |
| 15 | Q8 | *Enterobacter cloacae* | Sugarcane root | 0.00% |
| 16 | Q9 | *Bacillus amyloliquefaciens* | Sugarcane root | 3.33% |
| 17 | Q10 | *Bacillus amyloliquefaciens* | Sugarcane root | 0.00% |
| 18 | Q11 | *Bacillus amyloliquefaciens* | Sugarcane root | 0.00% |
| 19 | B9 | *Bacillus subtilis* | Sugarcane root | 0.00% |
| 20 | T1 | *Paenibacillus polymyxa* | Tobacco leaves | 0.00% |
| 21 | T2 | *Bacillus megaterium* | Tobacco leaves | 0.00% |
| 22 | T3 | *Bacillus amyloliquefaciens* | Tobacco leaves | 0.00% |
| 23 | Pb | *Pseudomonas* | / | 96.67% |
| 24 | Th-B | *Trichoderma harizanium* | / | 93.33% |
| 25 | Y2 | *Bacillus amyloliquefaciens* | Wheat rhizosphere soil | 100.00% |
| 26 | L1-1 | *Bacillus subtilis* | Citrus leaves | 0.00% |
| 27 | L1-2 | *Bacillus subtilis* | Citrus leaves | 0.00% |
| 28 | L1-5 | *Bacillus subtilis* | Citrus leaves | 6.67% |
| 29 | S1-2 | *Bacillus velezensis* | Citrus stem | 0.00% |
| 30 | S1-4 | *Bacillus velezensis* | Citrus stem | 0.00% |
| 31 | O42 | *Bacillus amyloliquefaciens* | Citrus leaves | 0.00% |
| 32 | YS3 | *Bacillus subtilis* | Citrus leaves | 6.67% |
| 33 | WT-1 | *Curtobacterium* sp. | Citrus leaves | 0.00% |
| 34 | WT-2 | *Curtobacterium* sp. | Citrus leaves | 0.00% |
| 35 | WT-3 | *Curtobacterium* sp. | Citrus leaves | 0.00% |
| 36 | WH-2 | *Bacillus subtilis* | Citrus fruit | 0.00% |
| 37 | WH-3 | *Bacillus subtilis* | Citrus fruit | 3.33% |

- Inhibition rate was calculated by (Number of inhibition zones/Total number of colonies ×100)
